# Evaluating the Effects of Environmental Conditions on Sensed Parameters for Green Areas Monitoring and Smart Irrigation Systems

**DOI:** 10.3390/s21062255

**Published:** 2021-03-23

**Authors:** Pedro V. Mauri, Lorena Parra, Salima Yousfi, Jaime Lloret, Jose F. Marin

**Affiliations:** 1 Instituto Madrileño de Investigación y Desarrollo Rural, Agrario y Alimentario (IMIDRA), Finca “El Encin”, A-2, Km 38, 2, Alcalá de Henares, 28805 Madrid, Spain; pedro.mauri@madrid.org (P.V.M.); loparbo@doctor.upv.es (L.P.); salima.yousfi@madrid.org (S.Y.); 2 Instituto de Investigación para la Gestión Integrada de Zonas Costeras Universitat Politècnica de València, 46730 Valencia, Spain; 3 Area Verde MG Projects SL. C/Oña, 43, 28933 Madrid, Spain; jmarin@areaverde.es

**Keywords:** hourly variation, canopy temperature, soil temperature, soil moisture, vegetation indexes, turfgrass monitoring

## Abstract

The irrigation of green areas in cities should be managed appropriately to ensure its sustainability. In large cities, not all green areas might be monitored simultaneously, and the data acquisition time can skew the gathered value. Our purpose is to evaluate which parameter has a lower hourly variation. We included soil parameters (soil temperature and moisture) and plant parameters (canopy temperature and vegetation indexes). Data were gathered at 5 different hours in 11 different experimental plots with variable irrigation and with different grass composition. The results indicate that soil moisture and Normalized Difference Vegetation Index are the sole parameters not affected by the data acquisition time. For soil moisture, the maximum difference was in experimental plot 4, with values of 21% at 10:45 AM and 27% at 8:45 AM. On the other hand, canopy temperature is the most affected parameter with a mean variation of 15 °C in the morning. The maximum variation was in experimental plot 8 with a 19 °C at 8:45 AM and 39 °C at 12:45 PM. Data acquisition time affected the correlation between soil moisture and canopy temperature. We can affirm that data acquisition time has to be included as a variability source. Finally, our conclusion indicates that it is vital to consider data acquisition time to ensure water distribution for irrigation in cities.

## 1. Introduction

Reaching sustainability and the efficient use of resources is one of the major pillars in smart cities. The use of different technologies, such as Wireless Sensor Networks (WSN) or remote sensing, to achieve these goals is a promising option. The efficient management of water is one of the Sustainable Development Goals. We can find several examples in which WSN and remote sensing benefits are reported [1,2]. In smart cities, we can identify different water uses, and all of them must be adequately managed to accomplish sustainability. Despite the fast growth of the above-mentioned technologies, they cannot be easily applied in some specific areas or monitoring certain parameters, and manual data gathering is still necessary. One example is plant stress monitoring in gardens to adjust irrigation [3].

The monitoring of some parameters, such as Soil Moisture (SM) or Soil Temperature (ST), is efficiently conducted with sensors in a WSN [4]. Nonetheless, other parameters related to grass are essential and must be considered to optimise irrigation. The Normalized Difference Vegetation Index (NDVI), the Canopy Temperature (CT), and other plant indexes, such as Green Area (GA), and Greener Area (GGA), are some of them [5,6]. Those parameters can be measured remotely, with specific devices, and offer more accurate information for smart irrigation systems. Therefore, for optimal monitoring, we must combine WSN with remote sensing (RS).

The use of RS to obtain data based on drones or smart vehicles used in gardening can be integrated with the data gathered from the WSN [7]. Nonetheless, the use of RS includes a new variable that must be considered in the systems, the data acquisition time (DAT). The DAT refers to the precise time at which the data is gathered. This information is crucial to jointly interpret the information gathered by the WSN with the data from the RS. The importance of DAT is due to the effect of environmental conditions on the gathered data. The principal environmental conditions that might affect the gathered data are solar exposure, which affects the light conditions and ambient temperature (AT), humidity, and wind. Those environmental conditions can suffer rapid changes along the day, affecting the measured parameters in two ways. The first is that the change in environmental conditions might modify the monitored parameters. For example, when the monitored parameter is the temperature of a surface exposed to the sun. On the other hand, environmental parameters might modify the lecture of the parameters without modifying the parameter itself. This is common in parameters measured or calculated using radiometric data, which can be affected by differences in the light conditions.

Focusing on smart cities and the smart irrigation systems, we can find that some of the monitored variables are clearly affected, while others are not so clear. In some cases, as the CT, it is clear that the variation on solar exposure along the day will produce a variation on the CT. Nonetheless, there are variables such as NDVI, GA, and GGA, for which it is not clear if the variation of solar exposure will have a clear impact on its measurement. While NDVI measures the infrared and red regions of the electromagnetic spectrum, other vegetation indexes measure only the visible spectrum. Among those indexes, the GA and GGA are used to monitor the plant status [5].

Therefore, it is necessary to detail the measured parameters’ expected variation along the day, especially from morning to midday. This is particularly relevant for smart irrigation systems. Determining which parameters are affected by the DAT and, if possible, establish a relation between atmospheric conditions and gathered data is crucial to ensure proper irrigation management.

The aim of this paper is to evaluate the effects of different environmental conditions on six parameters gathered at different DAT to determine which parameter is less affected by environmental conditions and is a better indicator of plant performance. We measure those six parameters, including SM, ST, CT, and vegetation indexes (NDVI, GA, and GGA), in a period of five hours in 11 different plots. The data is gathered during one morning to maximise the variation of environmental conditions. Then, the data is analysed to examine the effect of those changing environmental conditions in each one of the monitored parameters. As a result, we will determine which of the monitored parameters in this assay is less affected by the environmental conditions and can be the better indicator for defining the irrigation needs in smart gardens.

The rest of the paper is structured as follows; Section 2 provides a framework of the state of the art. The materials and methods are described in Section 3. Section 4 portrays the results of the different measures. The discussion of our findings is carried out in Section 5. Finally, Section 6 summarises our conclusions.

## 2. State of the Art

In this section, related work is described. This section is divided into two different subsections. The first one is focused on studying the effect of DAT in data series in cities and green areas/farming areas, mainly focused on the hourly variation of data. The second section seeks to outline the state of the art regarding grass and their irrigation-need monitorisation.

### 2.1. Hourly Changes and the Effect of Environmental Conditions on Monitoring Variables

Some variables used to characterise or monitor cities and agricultural areas have high variability over short periods. Those fast changes can be induced by natural processes changes, such as meteorology or changes from day to night, or affected by human interaction.

Following, we detail some cases in which a change in studied variables in cities is due to human interaction change. The most relevant and studied effect is the changes in pollution in cities. In [8], the authors studied the hourly concentration of particulate matter in Monroe County, NY, USA. They measured the concentration of PM in up to 50 points, and data were gathered each minute. Their results indicate that particulate matter is highly influenced by traffic. They identify lower values from 5 to 6 AM in all the studied points and two peaks at 12 PM and 6 PM. Temporal variation of urban heat island (UHI) was studied in [9]. Their results indicate that UHI can be used as an indicator or urban or rural areas. Nonetheless, the UHI changes along the day, the nighttime UHI intensity being much stronger than daytime UHI. There is even a period (from late morning to noon) in which there is a possibility or inverse UHI intensity.

On the other hand, there are parameters mostly influenced by meteorological factors or changes from day to night. In [10], the authors studied the changes in the distribution of spore trap at a university campus. They conclude that there are no statistically significant differences over the days, but there are hourly variations. Nonetheless, the authors indicate that hourly data should be interpreted with caution due to the high degree of uncertainty linked to those values. The encountered variation in hourly values between samplers was superior to the daily variation.

Focusing on the temporal variability of measured parameters in this study, Brocca et al. reported in [11] after measuring the SM in the Vallaccia catchment that the temporal variability was higher than the spatial variability. In their studied area, there are different types of soils (from gravel to clay soils) and different slopes. Their data include one year of samples, having 1 sample per day. They conclude that the deployment of a few sensors with a fine temporal resolution, at least one measure per hour or less, is the best option for SM monitoring at the catchment scale. In a similar study, Evett et al. [12] indicate that SM’s spatial variability was higher than spatial variability. The data used by Evett et al. was obtained in the Conservation and Production Research Laboratory at Bushland, TX (USA), after applying different irrigation regimes. The aim was to generate data that allows the comparison of two adjacent plots with different irrigation. They perform the measures on uniform Pullman soil in which winter wheat was cropped. One plot received 100% of irrigation and the other just 33%. The authors do not indicate the time interval between measures. Several authors indicate that the interaction of plant-soil might affect the temporal variability of SM. Chen and Hu indicate that the presence of crops reduces the temporal variability of SM [13]. Meanwhile, Hupet and Vanclooster [14] indicated that it might reduce the temporal stability of data. Therefore, there is no clear consensus on this topic.

Finally, regarding the variation in a plant, we can find some studies that evaluate the hourly changes in measurable parameters of plants. Wang et al. [15] measured the variation of plant temperature and vapour pressure deficit in maize plants every 30 min over three days in two periods of time. Their results indicate that the variation from a cloudy to clear day modifies the temporal variation. The authors reported a variety of plant temperature of up to 15 °C. The registered vapour pressure deficit goes from 0 to 4 kPa. The authors conclude that meteorology is the main factor affecting the changes in measured parameters. Ruiz et al. evaluate in [16] the hourly changes of litter moisture content in *Eucalyptus globulus*. Their results indicate that there is a variation in litter moisture content, the maximum content being at night. In contrast, the drying up effect of the sun reduces the moisture in a very short time. Another relevant example can be found in [17], where Xu and Wang showed a time series in a man-made forest. The authors combined three remote sensing sources to evaluate the changes in this forest. Thus, they have multiple images in a short period. Although this is an innovative idea, the remote sensing sources’ current resolution precludes its use in our experiments.

### 2.2. Relevant Parameters for Monitoring Irrigation Requirements in Turfgrass

Following, we focus on the selected parameters for monitoring the irrigation requirements for turfgrass. There are several proposals of systems to manage the irrigation of turfgrass. We summarise the different parameters used to define the irrigation need or plant stress.

The most basic systems are the ones that calculate the irrigation based on potential evapotranspiration (PET) in which no sensors are used. The most commonly used sensors are the ones that measure the SM, as the ones detailed in [18]. In [18], Grabow et al. compared two systems’ irrigation efficiency, one based on the PET and the other based on SM sensors. Their results showed that, on average, using SM sensors supposes a saving in water of about 50% of the PET-based system’s water. A second soil parameter is measured in [19] by Hong et al., the ST. In [19], the authors measured several parameters in small plots under different irrigation. They identify correlations between CT and some indexes such as NDVI and with the green cover. Nonetheless, a low correlation was found for CT and water content in the first year of the study and no correlation was found for the second year. Thus, the usability of ST is not entirely clear. Another relevant value to indicate the irrigation requirements is the CT, as indicated in [20] by Hong et al. Their results indicate that CT reflects plant stress before visible drought symptoms appear. They have 24 plots with different irrigation. They indicate that CT detects stress before NDVI and other commonly used indexes.

Focusing on indexes, NDVI has been used in previous studies [19,20]. Other examples can be found in [5,6] in which NDVI is used in combination with GA and GGA to evaluate plant drought stress, and high correlations were found.

## 3. Materials and Methods

In this section, we describe the location and environmental conditions of the studied area. First, we present the different monitored plots, detailing the grass species they contain, and their maintenance. Then, the monitored parameters and their relevance for smart irrigation systems of smart cities are defined. Finally, the methodology followed to gather the data for this paper is explained.

The area selected for this assay is located in the Mediterranean region of Spain, specifically in the eastern part of Madrid, in the facilities of IMIDRA. The climatic characteristics of this area can be found in [21].

### 3.1. Description of Monitored Plots of Turfgrass

In order to analyse the variation of monitored parameters without measuring the effect of specific turfgrass species, we monitored the variation of parameters in four grass combinations. This way, if the effects are higher or lower in any of the monitored combinations, they can be detected and considered for future studies. Therefore, four grass combinations, set in eleven experimental plots, were considered for the monitoring of the variables. The turfgrass mixtures included in this assay are detailed in Table 1. Those eleven plots include three repetitions of three mixtures (F+C, F+Z, and F+B) and two repetitions of one mixture (F+P+L). Each plot has an extension of 4.5 m^2^ (1.5 m × 3 m). The grass mixture (GM) was one of the evaluated sources of data variations.

The experimental plots were maintained over one year under regular irrigation, 100% of evapotranspiration. Although the plots were irrigated at 100% evapotranspiration, there was small differences between plots due to predominant winds. Therefore, we can identify that some plots received more water than the others. The precise amount of received water of each plot was controlled with a rain gauge, and these small differences will be considered in the analysis of the data. The irrigation received (IR) is the second source of variation evaluated. The plots were mowed once per week to maintain appropriate grass height.

### 3.2. Measured Parameters and Used Equipment

In this study, we included six parameters, which include the monitorisation of soil and plants. With regard to the soil, SM and ST were measured; for the measurement, we selected a TDR 350 FieldScout [22]. SM was measured as the volumetric water content in percentage with a precision of ±0.3%. ST was measured in °C with a precision of ±1 °C. The data of SM and ST were measured at 5 cm depth. In each plot, three measurements were performed to obtain the mean value. The measures were gathered, guaranteeing that they represent the entire plot, including the central and edge areas. The desired values of SM to have turfgrass with high quality is between 30 to 40 %. Since the soil in which we performed the measures in sandy loam soil, SM’s values can range from 5–10% to 40–45%.

**Figure 1:**
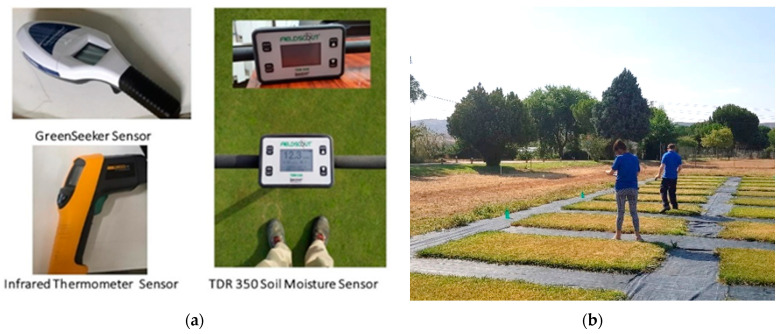
Pictures of (**a**) used devices and (**b**) data gathering process.

Concerning the plant, we measured CT using an infrared thermometer, the Fluke 561 [23], with a precision of ±1 °C. The CT increases as the water content of the plant decreases and its stress increases. When the plants have a high amount of water, their CT is lower and suffer fewer changes along the day due to water properties. Figure 1a shows the used devices. The scenario with the measurements is shown in Figure 1b.

On the other hand, vegetation indexes were evaluated. Regarding the NDVI, a handheld GreenSeeker [24] was used. The manufacturer does not offer the precision of this measure. This index can have values from 0 to 1. The higher the value, the greater the plant vigour. Finally, the GA and GGA have the same possible values as NDVI, from 0 to 1. Again, the higher the vegetation index value, the higher the vegetation vigour. For the calculation of GA and GGA, pictures of each plot were analysed with the BreedPix 0.2 free-access software. SONY DSC-W120 was selected to gather the pictures used for the GA and GGA obtention.

### 3.3. Methodology Implemented for the Data Gathering

To evaluate the effect of environmental conditions in the DAT of the aforementioned variables, we designed a protocol for gathering the data in the 11 pots. Since most of the maintenance tasks in urban gardens are performed during the morning, we planned our sampling period from 08:45 AM to 12:45 PM. Thus, we cover most of the regular hours at which the maintenance tasks are performed and register data with different solar exposure conditions. The measures were gathered on the 15 July 2020; the sun rose at 06:57 AM. The summary of environmental conditions at each DAT is shown in Table 2. DAT is the third source of variation in the gathered data.

Data were gathered each hour, and the data gathering has a duration of 15 min approximately. Three researchers worked in parallel to gather the data to minimise the time required for the data gathering. We started from plot 1 to plot 11. The parameters were monitored plot by plot, starting with the TDR (SM and ST), following with the NDVI, CT, and finally, the picture.

### 3.4. Methodology Implemented for the Data Analysis

Once the data is gathered, we analysed it to evaluate the effect of each one of the factors over the parameters. First of all, we identified the evaluated parameters and factors, detailing their units. We included the six variables (SM (%), ST (°C), CT (°C), NDVI, GA, and GGA) and three factors of variability sources (GM, IR (mm), and DAT (HH:MM)). To evaluate the effect of factors over the variables, a multifactorial ANOVA procedure was performed. The ANOVA procedure allows determining if a factor affects a variable based on this variable’s mean for each level of the factor (each GM, each IR or each DAT). The null hypothesis assumes that all groups’ means are the same; thus, the factor does not affect the variable. The alternative hypothesis assumes that each group’s means are different; thus, the factor affects the variable. This procedure is used to evaluate the effect of different factors, such as GM or IR, in [6]. Based on our factorial experimental design, we selected multifactorial ANOVAs to consider the multiple effects of the three included factors (GM, IR, and DAT) over each variable. The ANOVAs were calculated with Statgraphics Centurion XVI [25]. To represent the effect of each one of the studied factors, mean graphics were used. For those graphics, the Fisher Least significant difference (LSD) was selected. The *p*-values were used to evaluate the strength in which each factor affects the variables. *p*-values lower than 0.05 indicate that there is an effect of the studied factor over the variable.

After the effects of factors on the variables were analyzed to determine if the DAT makes impossible the comparison of measured variables or not, we evaluated the correlation between variables. In previous publications [5,6], it has been demonstrated that some of the measured variables present a high correlation, such as NDVI, GA, and GGA or between SM and CT. For these correlations, again, Stargraphics Centurion XVI is used. We selected simple regression models based, if possible, on linear regression, to keep the models as simple as possible. The *p*-values, correlation values, and R2 will indicate the strength of the correlations. Given the high variability over the units and their range of the monitored parameters, the mean absolute errors are not considered.

## 4. Results

In this section, we analyse the gathered data to evaluate if the DAT affects the monitored variables, which makes comparison of data with different DAT impossible. In addition, we evaluate if the DAT affects the existing and well-studied correlations among variables.

### 4.1. Analyses of Variance for Each One of the Monitored Parameters

In this subsection, we analyse the variance of each one of the monitored parameters considering the following factors: DAT, IR, and GM.

#### 4.1.1. Effects on SM

**Figure 2:**
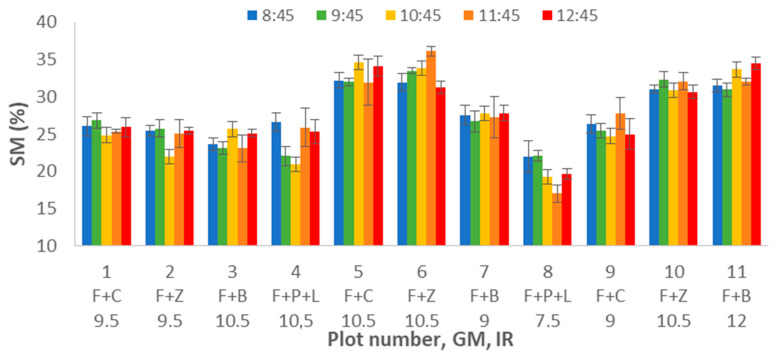
Mean values of SM for different plot numbers and DATs.

The SM data are summarised in Figure 2. Figure 2 presents the mean values of each plot (1 to 11) at different DATs (8:45 to 12:45), represented in different colours. The error bars represent the standard deviation. First of all, we can identify that apparently, there is no variation of SM related to the DAT. The lowest values of SM are found in plot 8. It is possible to note that some of the plots have greater SM values, mainly plot 5 and plot 6. The observed variation seems to be related to the small differences in irrigation between plots. The differences in soil uniformity explain that some plots have higher standard deviation than others and the general high values of standard deviation among the samples. These differences caused high spatial variability on the gathered data even in each plot.

**Figure 3:**
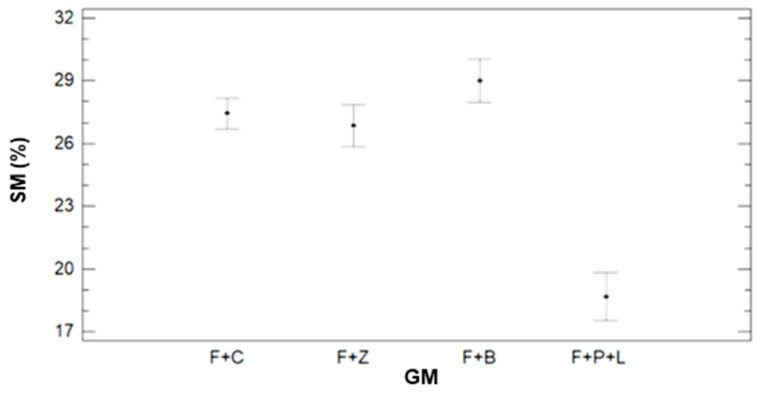
Mean values of SM and LSD intervals for multiple range tests of ANOVA for factor GM.

**Figure 4:**
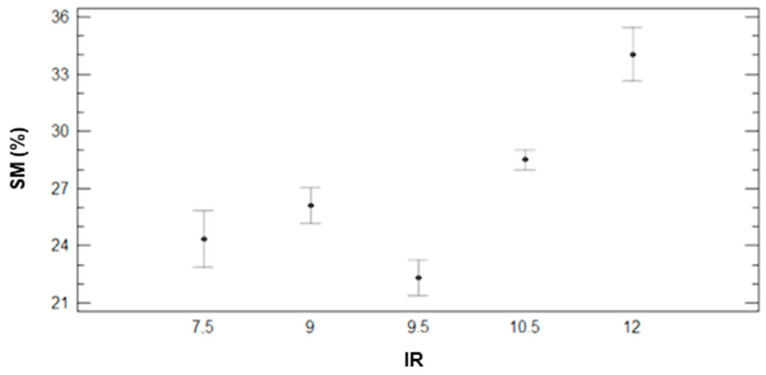
Mean values of SM and LSD intervals for multiple range tests of ANOVA for factor IR.

To properly evaluate the effects of DAT, GM, and IR on this variable, multifactorial ANOVA is performed. The results indicate that the data variability is caused by GM and IR, with *p*-values of 0.0000 for both factors. Meanwhile, the DAT does not affect gathered data of SM, with a *p*-value of 0.8722. Following, we show in Figure 3 and Figure 4, the variability of SM for GM (Figure 3) and IR (Figure 4). The error bars represent 95% of Fisher LSD intervals. In Figure 3, we can see that GM has a severe effect on the data of SM. There are big differences between F+P+L and the rest of GM. In previous work [6], we determined that this GM requires more water than the other ones. Therefore, the values of SM are lower in the plots with F+P+L, compared with other mixtures. The mean value of SM for F+P+L is 18.66%. With regard to the other GM (F+C, F+Z, and F+B), the three of them have similar SM values. According to the multiple range test of the ANOVA, we can identify two groups; the F+P+L composes the first group with the lower SM. The second group, characterised by higher SM, comprises the rest of the combinations, with mean values of 27.46%, 26.85%, and 29.02% for F+C, F+Z, and F+L.

On the other hand, Figure 4 represents the different SM values in the plots according to their IR. These data do not follow an increasing relation between IR and SM. The data obtained in plots with 9.5 mm of irrigations does not follow this trend. Nonetheless, excepting those plots, the ones that have received higher amounts of water are characterised by the highest SM values during the data gathering process.

#### 4.1.2. Effects on ST

**Figure 5:**
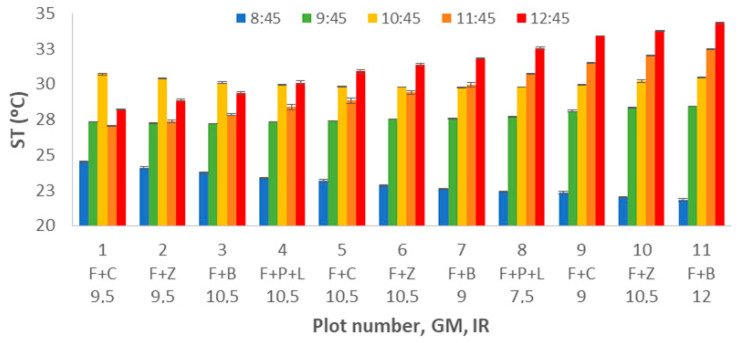
Mean values of ST for different plot numbers and DATs.

Now we deal with the variance found on the ST measured data. Figure 5 represents the mean values, and standard deviations for ST data gathered at different plots. First of all, and comparing with variance found in SM, we can affirm that in this case, the standard deviation of measured gathered in each plot and DAT is much lower. Although there is no clear tendency in the first plots, in plots 7 to 11, we can identify how the ST increases with the DAT. This lack of uniformity in the behaviour is caused by a significant ST measurement problem, the used sensor. The sensor included in the TDR requires several minutes to stabilise its value, and according to the place in which it was located, the temperature of the first plots might be higher or lower than the ST. Therefore, this increasing ST with the DAT is clearly identified in the last 4 plots, in which the time required to stabilise the ST sensor data is almost fulfilled. We can identify that in the measure at the first DAT, the ST decreases with the plots since the initial lecture of ST was higher than the real ST due to the FR being stored in an office. In the last DAT, the measured ST increases with the plot number since the office was colder than where the plots are located. This lack of uniformity in the measures is a major problem when ST data is gathered with this sort of equipment. It supposes a biased spatial variation in the generated data caused by this effect if several measures should be taken in a single location.

**Figure 6:**
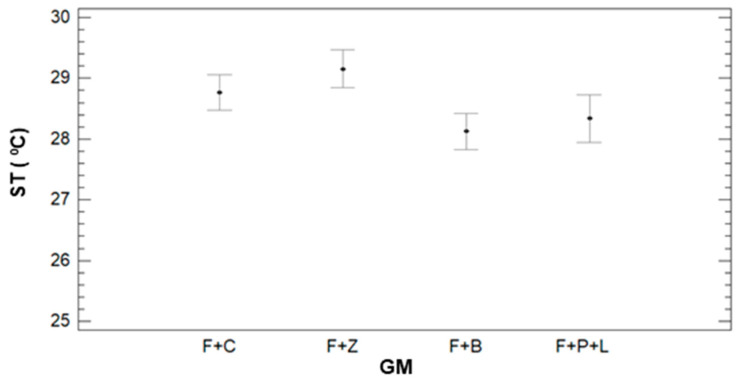
Mean values of SM and LSD intervals for multiple range tests of ANOVA for factor GM.

The multifactorial ANOVA is performed, and the results point out that all the variables have *p*-values lower than 0.05. Thus, the observed variability of the data is caused by GM, IR, and DAT. According to the ST variance for the different GM and the multiple range tests results, we can identify three groups (see Figure 6). The first group is composed of F+B and F+P+L with mean values of 28.12 °C and 28.34 °C. F+B and F+C compose the second group. Finally, F+C and F+Z form the last group with mean values of 28.76 °C and 29.15 °C. With regards to the IR, no clear tendency is observed between TS and IR. Therefore, these results are not included as a figure.

**Figure 7:**
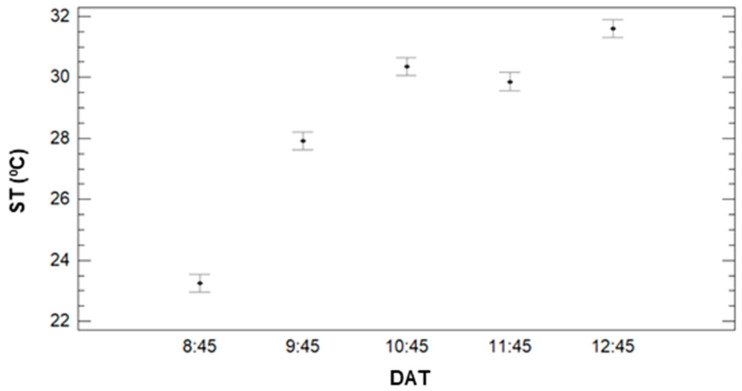
Mean values of SM and LSD intervals for multiple range tests of ANOVA for factor DAT.

Regarding the effect of DAT, several differences are observed in the ST data, see Figure 7. The data of ST clearly increases with the DT, as previously observed in Figure 5. With the multiple range tests, we identify four groups: the group with the lowest ST, the one composed of DAT data = 8:45. The mean value of ST of this group is 23.25 °C. The second group is formed by DAT = 9:45, with a mean value of 27.91 °C. Data measured in the third and fourth DAT, with mean values of 29.86 °C and 30.36 °C, are included in the thirds group. Finally, the data from DAT = 12:45 is classified as the fourth group, with a mean value of 31.59 °C.

#### 4.1.3. Effects on CT

**Figure 8:**
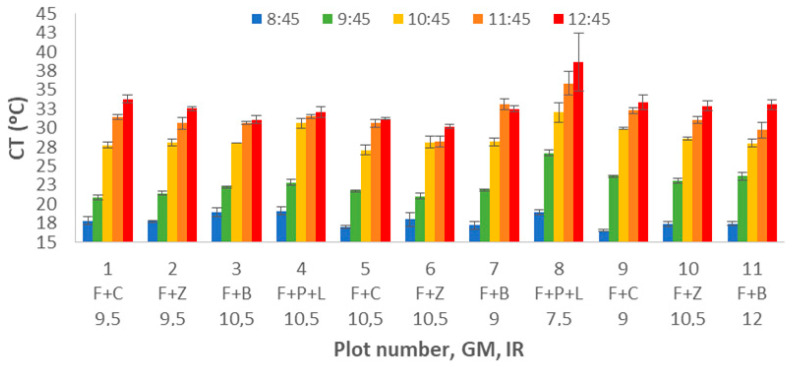
Mean values of CT for different plot numbers and DATs.

The analysis of CT data is presented in Figure 8. Several similarities can be found between ST and CT data. We clearly identify the increasing pattern with the DAT. Nonetheless, as in this case, temperature measurement is based on the infrared measure, the measure is not affected by the temperature of the sensor, and no time is needed to have a stable measurement. Therefore, the effects are more evident with this data. The lowest measured data was gathered at plot 9 in the first DAT; CT was 16.5 °C. The highest value was measured at plot 8 during the last measuring time, 35.9 °C.

**Figure 9:**
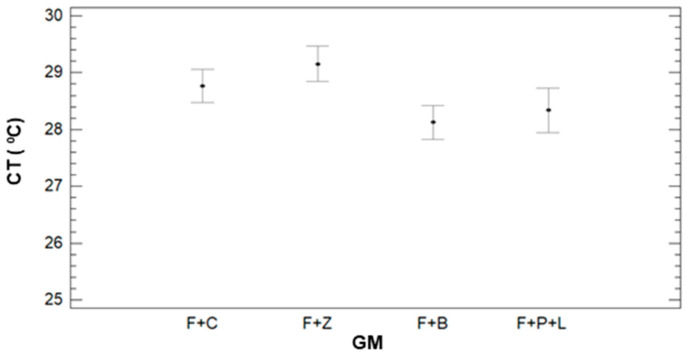
Mean values of CT and LSD intervals for multiple range tests of ANOVA for factor GM.

To evaluate the impact of each factor in the variance of CT, we analyse the results of multifactorial ANOVA, and see the mean values and LSD intervals in Figure 9. As for the ST, all the factors have a *p*-value lower than 0.005. The first described factor is GM. We can identify that the CT for F+C, F+Z, and F+B are almost equal and considered a single group. The mean values for these GM are 26.82 °C (F+Z), 26.89 °C (F+B), and 26.90 °C (F+C). The other group is composed of F+P+L with a mean value of 28.22 °C.

**Figure 10:**
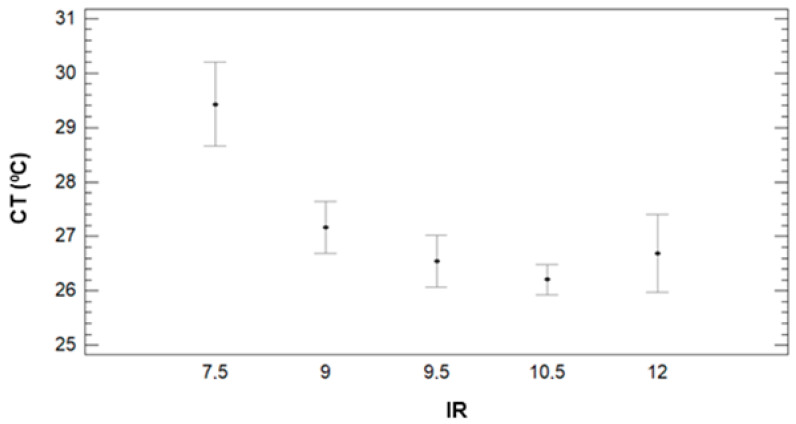
Mean values of CT and LSD intervals for multiple range tests of ANOVA for factor IR.

**Figure 11:**
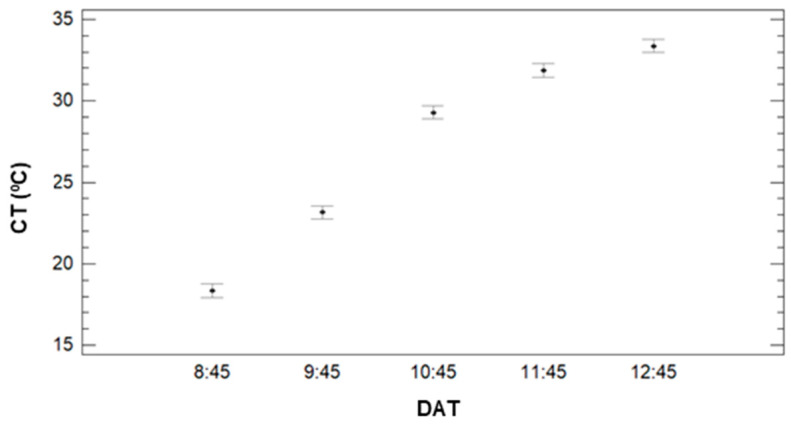
Mean values of SM and LSD intervals for multiple range tests of ANOVA for factor DAT.

Concerning the effect of IR, see Figure 10, we can clearly identify CT’s decrease when the IR increases. The multiple range tests created three different groups. With lower CT values, the first group is composed of 9.5 to 12 mm of irrigation with Ct values of 26.5 °C (IR = 9.5), 26.2 °C (IR = 10.5), and 26.7 °C. Last, the effect of DAT is described in Figure 11. It is possible to identify the increasing pattern, which was identified in Figure 8. According to the different created groups with multiple range test, five groups are created. Each group contains a single DAT, and the groups confirm that the CT increases with the DAT. Mean values are 18.34 °C (8:45), 23.15 °C (9:45), 29.29 °C (10:45), 31.89 °C (11:45), and 33.37 °C (12:45).

#### 4.1.4. Effects on NDVI

**Figure 12:**
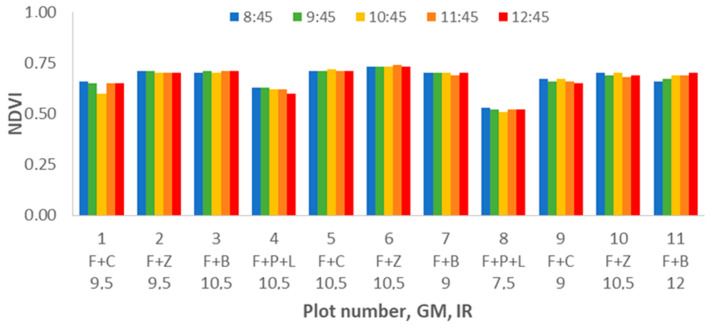
Mean values of NDVI for different plot numbers and DATs.

The results of NDVI indicate very stable data along all the studied period. We can identify differences among the measured plots due to GM and IR, but the differences are not related to the DAT. The highest NDVI values are registered in plot 6, F+Z and 10.5 mm of IR. In this plot, the value of NDVI reached 0.763 at DAT = 11:45. The lowest values are always gathered in plot 8 (F+P+L with IR = 7.5), the lowest at 0.51 being measured at DAT = 10:45. As shown in Figure 12, no effect of DAT is found according to the gathered data.

**Figure 13:**
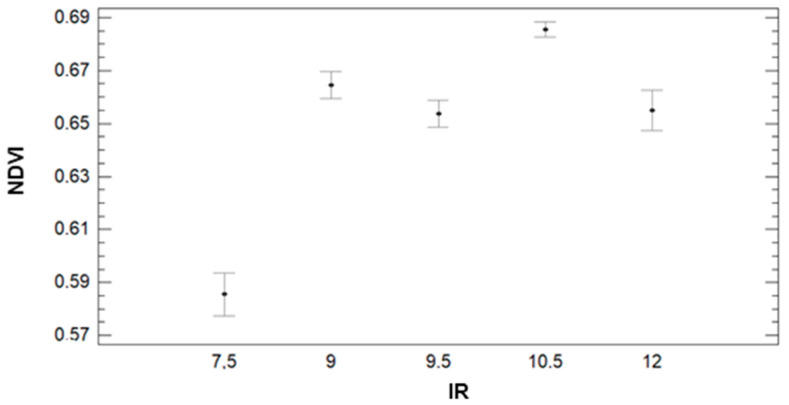
Mean values of NDVI and LSD intervals for multiple range tests of ANOVA for factor IR.

**Figure 14:**
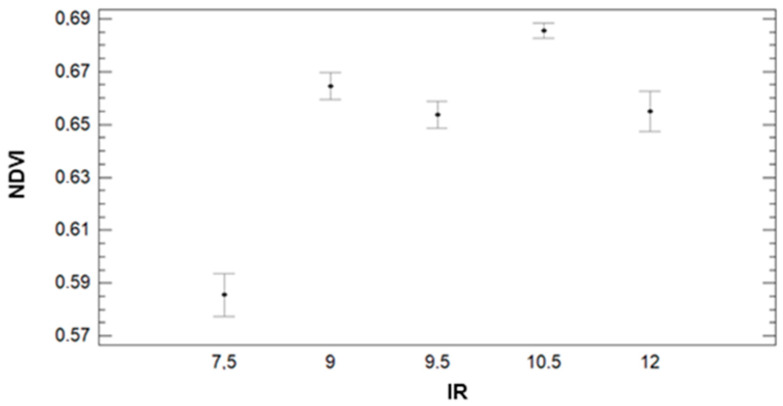
Mean values of NDVI and LSD intervals for multiple range tests of ANOVA for factor GM.

Regarding the results of ANOVA analyses, we can identify an apparent trend between NDVI and IR. The NDVI is lowest for the lowest IR value and increases considerably for the rest of the IR values (see Figure 13). Concerning the multiple range tests, for IR = 7.5, the average value of NDVI is 0.59, and those data compose the group with the lowest NDVI values. The second group comprises data from IR = 9, 9.5, and 12 with average values of NDVI of 0.66, 0.66, and 0.65, respectively. Finally, the group with the highest NDVI (average value of 0.69) is formed by data of plots with an IR of 10.5. Finally, with regard to Figure 14, the GM has a great effect on the NDVI. This effect is clearly indicated in the multiple range tests. The GM with the lowest NDVI values, the first group, is the F+P+L with an average of 0.58. The second group is composed of F+C, with an average NDVI of 0.66. Finally, F+B and F+Z have average NDVI values of 0.68 and compose the third group.

#### 4.1.5. Effects on GA

**Figure 15:**
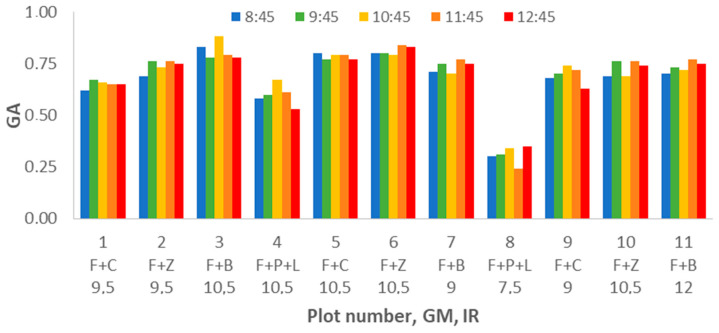
Mean values of GA for different plot numbers and DATs.

We analyze the variation on the second vegetation index, the GA, measured during the study. According to the results shown in Figure 15, we can clearly identify the decrease of GA for the plots composed of F+P+L compared with the other GM. Contrary to the results found for NDVI, in GA, we can identify the effect of DAT. Most of the plots have their lower GA value at DAT = 8:45 and their maximum GA at DAT = 9:45 or DAT = 10:45. The results of analysing the gathered data with multifactorial ANOVA indicate that DAT, GM, and IR are the factors that explain the variation in data. The *p*-values for GM and IR are 0.0000; meanwhile, for DAT, the *p*-value is 0.0198.

**Figure 16:**
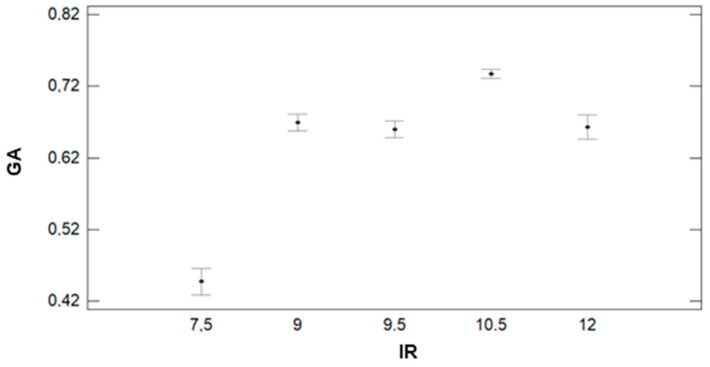
Mean values of GA and LSD intervals for multiple range tests of ANOVA for factor IR.

**Figure 17:**
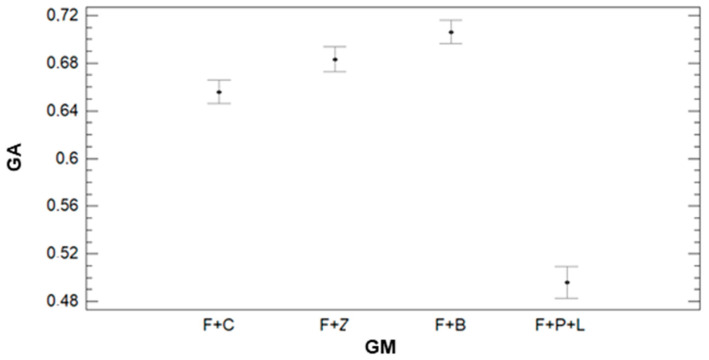
Mean values of GA and LSD intervals for multiple range tests of ANOVA for factor GM.

In Figure 16, we can see that IR has a clear effect on GA. According to the results of the ANOVA, we can identify three groups. The first groups with the lowest GA values are composed of the lowest irrigation plots (IR = 7.5). This group is characterised by an average value of 0.45. The second group, formed by plots with IR = 9, 9.5, and 12 mm, has an average GA value of 0.65 (9 mm) and 0.66 (9.5 and 12 mm). The last group is composed of plots with IR = 10.5 mm. In this group, the average GA value is 0.74. The effect of GM on the GA can be seen in Figure 17. We can find big differences in GA values between F+P+L and the rest of GMs, as shown for the other parameters. The values of GA are lower for the plots with F+P+L compared with GMs. The mean value of GA for F+P+L is 0.50, and this GM composes one of the four groups defined by the multiple range tests. The F+C composes the second group, with an average GA of 0.66. The third group is formed by F+Z, which has 0.68 as the average GA value. F+B composes the last group, with the highest GA value. The value of GA for F+B is 0.71, representing an increment of 42% compared with the GM of F+P+L.

**Figure 18:**
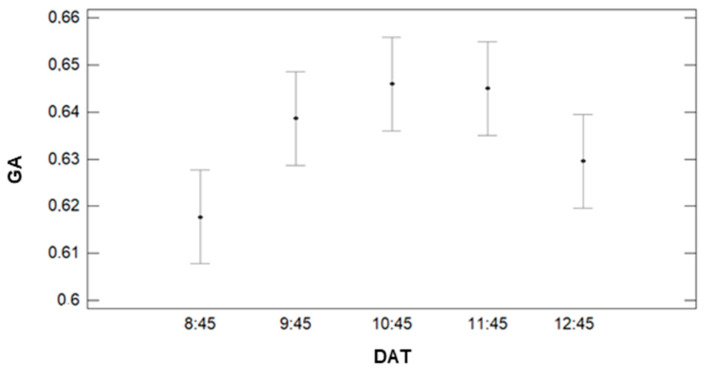
Mean values of GA and LSD intervals for multiple range tests of ANOVA for factor DAT.

Finally, we analyse the effect of DAT on the gathered data of GA (see Figure 18). For this factor, the results of the multiple range tests pointed out the creation of two groups. The first group comprises of DAT = 8:45 and 12:45, with average values of 0.62 and 0.63. The second group comprises of DATs of 9:45, 10:45, 11:45 and 12:45 with values of 0.64, 0.64, 0.65, and 0.63, respectively. Thus, we can affirm that for GA gathering, we can compare values taken in the central part of the morning (9:45 to 12:45), the period with the most similar values being from 10:45 to 11:45.

#### 4.1.6. Effects on GGA

**Figure 19:**
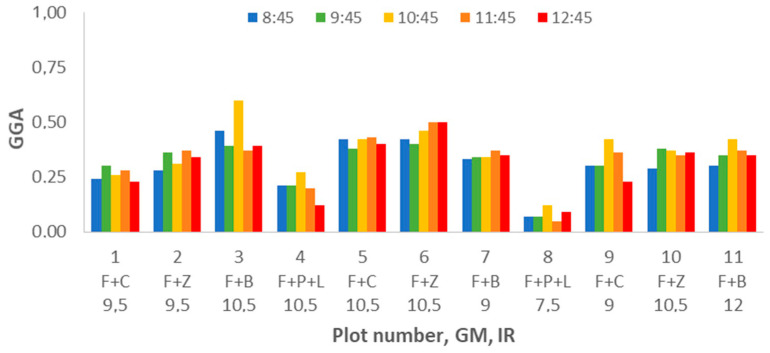
Mean values of GGA for different plot numbers and DATs.

Next, the last evaluated parameter, the GGA, is going to be analysed. The results of GGA for the different plots can be seen in Figure 19. In this graphic, we can identify some similarities with the GA data, Figure 15. In both cases, the plots composed of F+P+L present the lowest values. Compared with GA, the values of GGA are lower, as is expected due to the characteristics of each index. The case, plot, and DAT, with the highest GA value, correspond to the highest GGA value. The same trend is found for the case with the lowest value. Those cases are plot 3 and DAT 10:45 for the highest GA and GGA values, and plot 8 with DAT = 11:45 for the lowest value GA and GGA values. As in the previous cases, to analyse in depth the effect of the various factors on the variability of GGA, a multifactorial ANOVA is carried out. For GGA, the results of ANOVA indicate that the three factors have an effect on the variability of the data. The three factors have a *p*-value of 0.0000.

**Figure 20:**
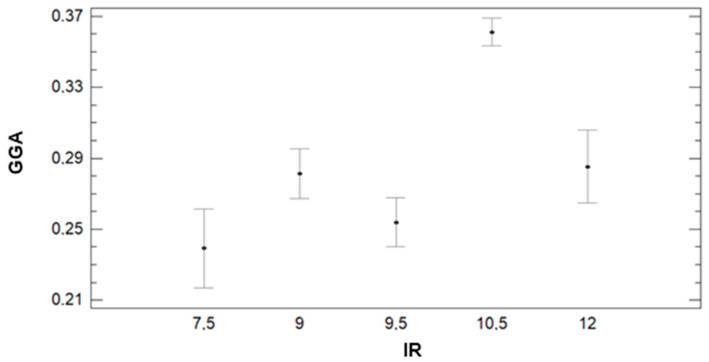
Mean values of GGA and LSD intervals for multiple range tests of ANOVA for factor IR.

Figure 20 presents the variability of GGA for the different IR regimens. Contrary to the tendency found with GA data, for GGA, the generated groups by the multiple range test have no apparent relation to the IR changes among the plots. In this case, three groups are identified. The group with the lowest GGA comprises plots with IR = 7.5, 9.5, and 12 mm with 0.24, 0.25, and 0.29 value for GGA. The second group is formed by plots with IR = 9 and 12 mm. The average GGA for plots of this group is 0.28 and 0.29, respectively. The plots with 10.5 mm of irrigation compose the last group, characterised by the highest value of GGA. The plots with IR = 10.5 have, on average, GGA value of 0.36. As mentioned before, those results are slightly different from the results of multiple range tests of GA for the IR factor.

**Figure 21:**
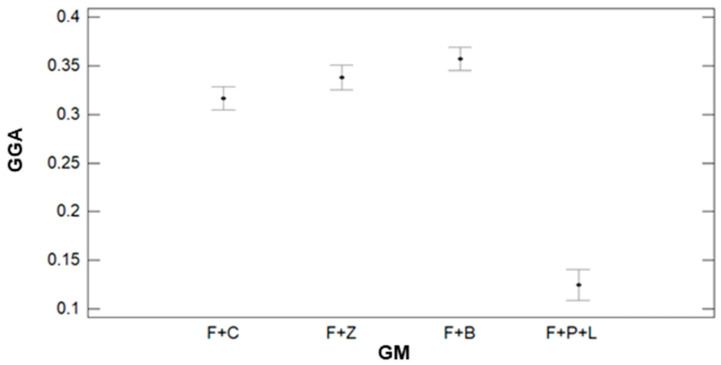
Mean values of GGA and LSD intervals for multiple range tests of ANOVA for factor GM.

Regarding the effect of GM on the variability of GGA, data is presented in Figure 21. This data is similar to the results found for the GA index. Despite GA, the multiple range tests identified four groups; in this case, only three groups are identified. The first group, characterised by the lowest GGA values, is composed of F+P+L. The average GGA value for this group is 0.12. The second group is formed by F+C, with an average GGA value of 0.32. Lastly, F+Z and F+B compose the last group, with average values of 0.34 and 0.36.

**Figure 22:**
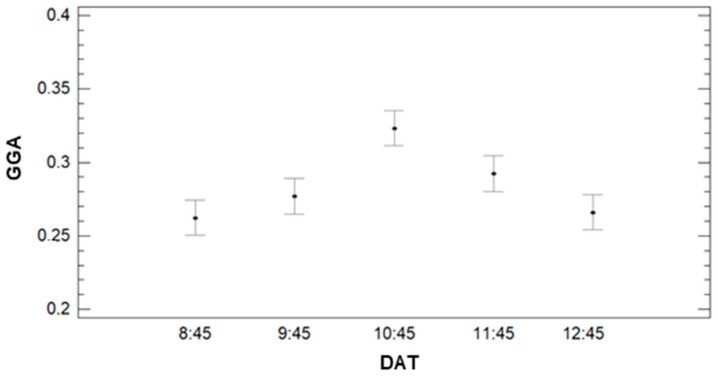
Mean values of GGA and LSD intervals for multiple range tests of ANOVA for factor DAT.

Finally, the effect of DAT on the variability of GGA is analysed in Figure 22. For this parameter, the multiple range test concludes that there are three different groups of data. The data gathered at DAT = 8:45, 9:45, and 12:45 composes the first group. The lowest values of GGA characterise this group. The average GGA value for data gathered at those DATs is 0.26, 0.27, and 0.28 for DATs 8:45, 12:45, and 9:45, respectively. The second group is formed by DAT = 9:45 and DAT = 11:45. The average GGA is 0.28 for DAT = 9:45 and 0.29 for DAT = 11:45. Finally, the last group comprises data gathered at DAT = 10:45, characterised by the highest GGA value. The average GGA for DAT = 10:45 is 0.32.

### 4.2. Correlation between Measured Parameters

In this subsection, we analyse the well-known correlation among the studied variables. To determine if the effect of DAT has disturbed those correlations, we are going to analyse the most common ones.

#### 4.2.1. NDVI-GA

**Figure 23:**
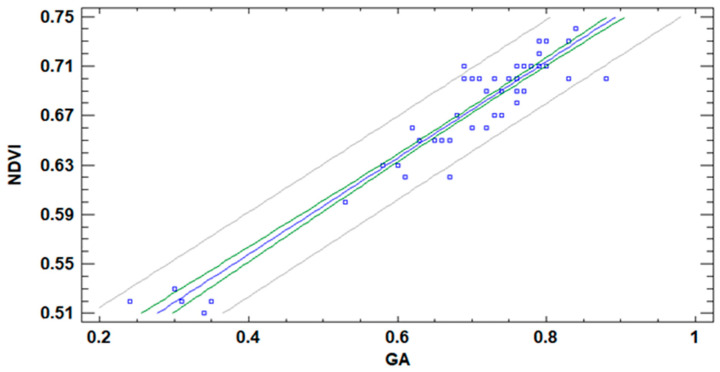
Correlation NDVI and GA indexes with data gathered at different DAT.

Several papers pointed out the correlation of NDVI with GA index in turfgrass [5,6]. Nonetheless, in those publications, the authors do not indicate if the data of both variables were collected at the same time or not. Considering that DAT has an effect on the GA index but not on the NDVI index, it is essential to confirm that the correlation between both variables can be done regardless of the DAT. Therefore, all the pairs of data from NDVI and GA, as well as a regression model, are included in Figure 23. In Figure 23, we can see the linear regression model and its confidence and prediction intervals. Although the linear regression model is not the one with the better correlation, the differences between the linear model and the one with the highest correlation are minimum (−0.96 and −0.95). Thus, to keep the model as simple as possible, we maintain the linear regression. The model presented in (1) has a Mean Absolute Error (MAE) of 0.013 and an R^2^ of 91%.

(1) NDVI=0.402+0.390×GA

Thus, we can affirm that, although the GA is affected by the DAT, the effect is small enough to allow the correlation between both indexes.

#### 4.2.2. NDVI-GGA

**Figure 24:**
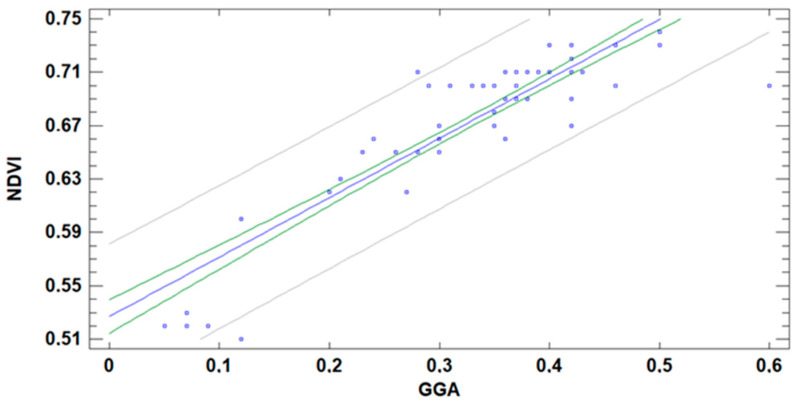
Correlation NDVI and GGA indexes with data gathered at different DAT.

The correlation between NDVI and GGA is also pointed out in several studies [5,6]. Figure 24 represents the pairs of data GGA and NDVI calculated based on the gathered data. Again, linear regression is selected to simplify the model. The model, presented in (2), has an MAE of 0.020 and R^2^ of 78%:

(2) NDVI=0.527+0.445×GA

Thus, we can affirm that, although the GGA is affected by the DAT, the effect is low enough to allow the correlation between both indexes. Nonetheless, the correlation is less accurate than the one obtained for GA and NDVI.

#### 4.2.3. SM-CT

**Figure 25:**
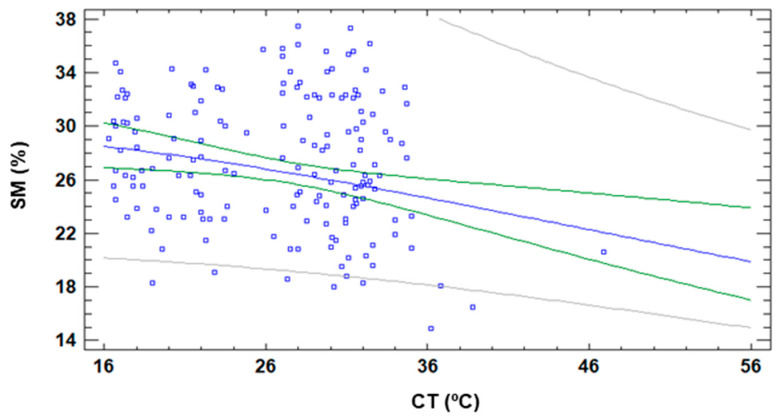
Correlation of SM and CT indexes with data gathered at different DAT.

Another well-known correlation is the one that models SM and CT; it is widely used when plant stress is monitored [6]. Considering that SM is not affected by the DAT and CT is strongly affected, it is necessary to evaluate if this correlation is maintained. Figure 25 displays the data of both variables as well as the most accurate regression model. Even though we selected the regression model with the highest correlation, the obtained correlation for SM and CT is very low. The correlation coefficient of the obtained model is 0.22. It is characterised by an MAE of 0.006 and an R^2^ of 5%. Thus, we can affirm that the effect of DAT in CT is so high that it invalidates SM’s correlation.

## 5. Discussion

### 5.1. Effect of DAT on Gathered Data and Its Importance to Compare Data

In this subsection, we will summarise the effect of the analysed factors on each of the monitored parameters and discuss the relevance of DAT to compare data.

First of all, a summary of the effect of each one of the factors on the monitored parameters can be seen in Table 3. We can identify that the parameter with a more significant effect on data variability is the IR, which affected all the evaluated parameters. This is a good result since all the evaluated parameters have the potential to be used as an indicator of the IR and the plant hydric stress. Nonetheless, we need to consider that IR’s effect does not follow the expected trend in some cases. This might be caused by the reduced number of repetitions in some of the IR and IR and GM’s combined effect in some of the plots.

Regarding GM, we can affirm that this factor affects all the parameters. Nonetheless, its effect on CT is lower than that of the other parameters. Contrary to the tendency observed with the effect of IR, GM’s effect in all the parameters follows the observed effects in previous studies [5,6]. F+P+L is the GM with the worst results in almost all the parameters (for ST, the lowest results are for F+B). Attending to the different parameters, in some cases, it is possible to identify two, three, or four groups. The SM allows the identification of two groups, the first one composed of F+P+L and the second one by the rest of the GM. The data of ST generates three groups, the first composed of F+B and F+P+L, the second by F+B and F+C, and the third by F+C and F+Z. With CT data, the multiple range tests formed three groups, the one formed by F+P+L having the highest CT. With regards to the vegetation indexes, all of them identify in a separated group the GM of F+P+L. The results of NDVI and GGA generated three groups, while the GA generated four groups. In all the cases, data of F+P+L represents the lowest value.

Concerning the DAT, we can affirm that it has a statistically significant effect on the temporal variability of ST, CT, GA, and GGA. Therefore, it is not possible to compare the data gathered at all the DATs in this study. Nonetheless, the hourly variability of SM and NDVI are null, which allow us to compare their values regardless of the DAT in which the data were gathered. Thus, we confirm that DAT and the hourly variability of data must be considered when data from different gardens are compared.

For the parameters affected by the DAT, we can identify different trends and some slots of time in which data can be compared. First of all, for the ST, the effect of temporal variability represents an increase of gathered data during almost all the studied period. In the period of 10:45 and 11:45, the STs slightly decrease instead of increasing. This is the unique period in which ST data can be compared. In the case of CT, a continually increasing tendency is observed, and no one slot of time allows the comparison of data. Concerning the GA and GGA indexes, both have the same trend, an initial increase in their values, with a maximum value at 10:45 and a posterior decrease. In the case of GA, this hourly variation allows the comparison of data gathered at 8:45 with data gathered at 12:45 and data gathered at 9:45 with 10:45, 11:45, and 12:45.

Thus, the sole problematic DAT is at 8:45. Therefore the gathering of GA must not be placed at the beginning of the morning. Finally, for GGA, we can find some slots of time that allows the comparison of data. For example, one slot is found with DAT 8:45, 9:45, and 12:45. The second slot of time is found for DATs of 9:45 and 11:45. A summary of these slots of time can be seen in Table 4. The different colours indicate the different slots of time. The DATs coloured in red indicate that there is no possible comparison. Slots in orange or in green allow the comparison between slots of the same colour. Some of the slots might be identified with two colours. Those changes might be explained according to the variation on meteorological data, mainly solar incidence and temperature, presented in Table 2.

### 5.2. Effect of DAT on Correlations

In this subsection, we will summarise the effect of DAT on the existing and well-known correlations and their relevance for posterior datasets creation.

First of all, a summary of the obtained correlations is given in Section 4 and other ones reported in related work can be seen in Table 5. We can see that the correlation NDVI-SM is not affected by the DAT since either of the two parameters is affected. The obtained correlation is similar to the one observed in [19], in which authors reported a correlation of 0.59.

Then, we identify two correlations with a low effect of DAT, the ones that related the indexes NDVI-GA and NDVI-GGA. Those correlations, especially the NDVI-GA, can be applied regardless of the DAT of data. Correlations NDVI-GA and NDVI-GGA are found in [5,6]. In [5], the authors found a correlation of 0.78 and 0.56 for NDVI-GA and NDVI-GGA, respectively. Those correlations were 0.64 and 0.35, respectively, in [6]. Thus, we can confirm that the effect of DAT is very low since, in our data, we have better correlations than the published ones.

Meanwhile, the correlations that include CT or ST are greatly affected by DAT and should not be applied with data gathered under different environmental conditions. Otherwise, it is possible to mix the effect of environmental conditions and the effect of monitored plant stress. It should be noted that the sole paper that reported this correlation found a value of −0.32 at a significance level of 0.05 [26]. Our results, although biased, show a similar trend. Regarding the ST, our results indicate that there is no correlation at all among the parameters. Nonetheless, in [19], the authors reported a correlation of 0.30.

### 5.3. Importance of the DAT and Temporal Variability for Monitorisation of Irrigation

The relevance of DAT when monitoring large areas is vital to ensure the proper distribution of water for irrigation. There are many cases in which water, as a scarce resource, must be handled to prioritise the irrigation of areas that have a higher need. Nonetheless, if data is not appropriately gathered and if we select a parameter affected by DAT, such as the CT, to define what area requires the irrigation, and data are obtained at different times slots, we might divert the water to plots with lower need. For example, a plot with high irrigation needs might have lower CT if it is gathered at the beginning of the morning than a plot without irrigation need which data is gathered at midday.

Therefore, for an appropriate irrigation management system, we should ensure that data gathered in the different plots can be compared. Here, we have different options. The most straightforward method is to select a parameter that can be compared, regardless of the DAT, such as SM or NDVI. Nevertheless, it is essential to indicate that the NDVI reflects plant vigour changes related to plant water requirements. Nonetheless, this change is only measured once the plant has started to suffer from hydric stress. The NDVI reflects the lack of irrigation in advanced status. Consequently, this indicator can be useful only in cases where we can assume that plants are kept under hydric stress for a period.

On the other hand, the SM is the parameter that can indicate the irrigation need in a shorter period since it measures the water content in the soil. Nonetheless, in this paper, we reported a huge variability of SM in each one of the plots. Many factors affect SM’s distribution in a soil portion, such as irrigation uniformity, soil texture uniformity, or even the punctual accumulation of salts. It must be considered that the variation of ionic content of the soil modifies its conductivity, and most of the sensors measure the SM based on soil conductivity. Thus, a sole measure of SM as proposed in several precision agriculture proposals cannot be accepted as reliable data of water content of a portion of the soil. Even though the problems are linked to a lack of uniformity, SM is the most direct and useful data for monitoring irrigation needs. In addition, the SM is a crucial parameter due to the inexistence of temporal variability encountered in this paper. It must be considered that the measurements gathered in this paper have been obtained before regular irrigation. The effect of irrigation or punctual rain will have a substantial effect on SM, invalidating data comparison. Nonetheless, this data must be extended to other periods of the year and with other climatic regions.

## 6. Conclusions

In this paper, we presented a problem related to the combination of WSN and remote sensing when monitoring large areas, and the effect of DAT on data variability. Due to the relevance and impact of data reliability, it is essential to evaluate which of the most-used irrigation management parameters is less affected by the DAT and should be prioritised to compare different green areas in large cities.

Our results indicate that just two out of the six evaluated parameters are not affected by the DAT—the SM and the NDVI. CT is the most affected parameter, and it is not possible to compare CT values gathered at different DAT. The hourly variation is so high that it does not allow the comparison of data. Meanwhile, ST, GA, and GGA are less affected by DAT, and there are specific slots of time in which the comparison of data gathered at different DAT is possible. For example, the hourly variation of ST is low from 10:45 am to 11:45 am. DAT also affects the well-known correlation between variables. Although some of them are still possible, such as NDVI-GA, others are not correlated. Some examples of correlation affected by the DAT are the ones that involve CT, such as CT-NDVI or CT-SM. For optimal irrigation management in large cities, good synchronisation of DAT will be required. Otherwise, it is possible to rely on parameters not affected by the DAT, such as SM, but it will be necessary to ensure that the spatial variation is considered in the deployment of the sensors. If NDVI is used for irrigation management, managers must consider that even though NDVI reflects drought stress regardless of the DAT, it is not an early indicator.

In future work, we plan to increase the gathered data, including data every 30 min and gather data in other moments of the year to evaluate if in winter, when there are fewer temperature changes, there is more temporal stability on the data. In addition, we plan to include new parameters, such as canopy temperature depression, more vegetation indexes, and thermal images. Finally, we plan to use fixed SM sensors in some of the plots to reduce the spatial variability in our measurements.

## Figures and Tables

**Table 1:** Details of the grass combination included in our study.

Id	Turfgrass Species	Acronym	C3/C4	No of Plots
1	*Festuca arundinacea* and *Cynodon actylon*	F+C	C3 & C4	3 (1, 5, & 9)
2	*Festuca arundinacea* and *Zoysia japonica*	F+Z	C3 & C4	3 (2, 6, &10)
3	*Festuca arundinacea* and *Buchloe dactyloides*	F+B	C3 & C4	3 (3, 7, &11)
4	*Festuca arundinacea*, *Poa pratensis*, *Lolium perenne*	F+P+L	All C3	2 (4 & 8)

**Table 2:** Summary of environmental conditions for each DAT.

DAT (HH:MM)	Sun Altitude (°)	Temperature (°C)	Relative Humidity (%)	Wind (km/h)	Incident Radiation (W/m^2^)
8:45	18.44	18.2	47.51	3.48	240.4
9:45	29.57	21.1	39.62	3.52	451.9
10:45	40.82	23.5	35.92	3.32	423.7
11:45	51.48	25.88	33.66	3.05	746.8
12:45	62.21	27.82	30.37	1.48	883.0

**Table 3:** Summary of the *p*-value for multifactorial ANOVAs for each monitored parameter and different factors.

Parameter	GM	IR	DAT
SM	0.0000	0.0000	0.9264
ST	0.0062	0.0000	0.0000
CT	0.0416	0.0000	0.0000
NDVI	0.0000	0.0000	0.7690 ^1^
GA	0.0000	0.0000	0.0198
GGA	0.0000	0.0000	0.0000

^1^*p*-value obtained if 95% of confidence.

**Table 4:** Summary of slots of time that allow the comparison of data for each variable. The green and orange colors indicate the possibility of comparing the data. The red colour indicates that there is no possibility to compare data.

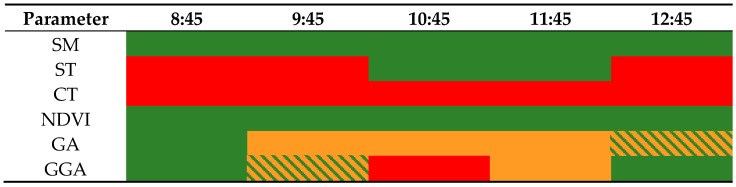

**Table 5:** Summary of correlations and the effect of DAT.

Parameters	Correlation	R^2^	Effect of DAT	Correlation Reported in:
NDVI-GA	0.95	91%	Very Low	[5,6]
NDVI-GGA	0.88	78%	Very Low	[5,6]
NDVI-SM	0.61	37%	Not affected	[19]
NDVI-ST	−0.0	0.1%	Extremely affected	[19]
NDVI-CT	−0.23	5%	Very High	[26]
CT-SM	0.22	5%	Very High	[6]

## Data Availability

The data presented in this study are available on request from the corresponding author.
